# Redifferentiation therapy in unresectable or metastatic radioactive iodine refractory thyroid cancer: an International Thyroid Oncology Group statement

**DOI:** 10.1016/S2213-8587(25)00064-6

**Published:** 2025-04-30

**Authors:** Sophie Leboulleux, Laura Boucai, Naifa Busaidy, Cosimo Durante, James A Fagin, Sasan Fazeli, Andrew G Gianoukakis, Bryan R Haugen, Hyunseok Kang, Bhavana Konda, Theodore W Laetsch, Laura Locati, Mabel Ryder, Christine Spitzweg, Francis P Worden, Lori Wirth, Alan Ho

**Affiliations:** Department of Medicine, Endocrinology and Diabetology Service, Geneva University Hospitals and Univerisity of Geneva, Geneva, Switzerland (Prof S Leboulleux MD PhD); Department of Medicine, Endocrinology Service, Memorial Sloan Kettering Cancer Center, New York, NY, USA (L Boucai MD); Department of Endocrine Neoplasia and Hormonal Disorders, The University of Texas MD Anderson Cancer Center, Houston, TX, USA (N Busaidy MD); Department of Translational and Precision Medicine, Sapienza University of Rome, Rome, Italy (Prof C Durante MD); Department of Medicine (Prof J A Fagin MD) and Human Oncology and Pathogenesis Program (Prof J A Fagin), Memorial Sloan Kettering Cancer Center, New York, NY, USA; Department of Clinical Diabetes, Endocrinology & Metabolism, City of Hope Comprehensive Cancer Center, Duarte, CA, USA (S Fazeli MD); Division of Endocrinology, Diabetes and Metabolism, David Geffen School of Medicine at UCLA Harbor-UCLA Medical Center, Los Angeles, CA, USA (A G Gianoukakis MD); Division of Endocrinology, Metabolism and Diabetes, University of Colorado School of Medicine, Aurora, CO, USA (Prof B Haugen MD); Department of Medicine, University of California at San Francisco, San Francisco, CA, USA (H Kang MD); Division of Medical Oncology, Department of Internal Medicine, The Ohio State University Comprehensive Cancer Center, Columbus, OH, USA (B Konda MD); Center for Childhood Cancer Research, Children’s Hospital of Philadelphia, Philadelphia, PA, USA (T W Laetsch MD); Medical Oncology Unit, Istituti Clinici Scientifici Maugeri IRCCS, Pavia, Italy (L Locati MD); Division of Endocrinology, Diabetes, Metabolism, and Nutrition, Mayo Clinic, Rochester, MN, USA (M Ryder MD); Department of Internal Medicine IV, LMU Munich, Germany (Prof C Spitzweg MD); Division of Endocrinology, Diabetes, Metabolism and Nutrition, Mayo Clinic, Rochester, Minnesota, USA (Prof C Spitzweg); Department of Internal Medicine, Division of Hematology and Oncology, University of Michigan Rogel Cancer Center, Ann Arbor, MI, USA (F Worden, MD); Harvard Medical School, Boston, MA, USA (Prof L Wirth MD); Center for Head and Neck Cancers, Massachusetts General Hospital, Boston, MA, USA (Prof L Wirth); Department of Medicine, Weill Cornell Medicine and New York Presbyterian Hospital, New York, NY, USA (A L Ho MD PhD); Head and Neck Medical Oncology Service, Department of Medicine, Memorial Sloan Kettering Cancer Center, New York, NY, USA (A L Ho)

## Abstract

In patients with follicular cell-derived thyroid cancer that have distant metastases and no iodine uptake, redifferentiation—ie, the restoration of tumoural ^131^I uptake with systemic therapy—is now possible. The use of mitogen-activated protein kinase (MAPK) inhibitors for a short period of time before the administration of high activity ^131^I shows promising results with iodine uptake restoration and tumour response. Redifferentiation has been used in patients with *BRAF*-mutated and *RAS*-mutated tumours in prospective trials and in the case of patients with *RET* or *NTRK* fusions. The iodine uptake restoration ranges from 33% to 95%, and tumour response rates from 11% to 80%. There is substantial variability between trials with regards to inclusion criteria, duration of redifferentiation drug therapy, activity of radioactive iodine, and use of dosimetry. Randomised studies are missing to clearly establish the effectiveness and applicability of redifferentiation. Thus, long-term studies are needed to establish the most effective redifferentiation protocols. The objectives of this Review are to: (1) provide a comprehensive review of the available results from prospective trials and case reports, including results regarding the restoration of radioiodine uptake and treatment efficacy (morphological and biological); (2) describe the differences in redifferentiation trial design between studies and discuss their potential impact on treatment efficacy; (3) describe the implications and limitations of dosimetry; and (4) outline the key questions to be addressed in future redifferentiation trials.

## Introduction

Distant metastases of follicular cell-derived thyroid cancer showing radioiodine uptake are treated with a high activity of radioiodine (^131^I). However, two-thirds of patients with metastatic lesions are, or become, radioiodine refractory (RAIR) during the course of their disease, primarily due to loss of cell differentiation leading to impaired iodine uptake and retention, or due to intrinsic radioresistance.^1^ Activation of the mitogen-activated protein kinase (MAPK) pathway is largely responsible for the loss of expression of proteins involved in iodine metabolism and is mainly driven by genetic alterations involving *BRAF*, *RAS*, *NF1*, *NTRK*, or *RET* genes that occur in the majority of RAIR thyroid cancers.^2–4^

Redifferentiation—ie, the restoration of tumoral ^131^I uptake with systemic therapy—was historically attempted with romidepsin and sorafenib, but failed to show significant clinical impact in patients.^5,6^ 13-*cis*-retinoic acid (isotretinoin) showed restoration of radioactive iodine (RAI) uptake in 17% to 33% of the patients; however, it was insufficient to induce tumour response after ^131^I therapy.^7–9^ Lithium was also associated with increased uptake and residence time of RAI in metastatic lesions.^10^ Unfortunately, overall survival and progression free survival did not differ between cohorts receiving or not receiving lithium as pretreatment to RAI.^11^ Digoxin has also been tested, but failed to show significant RAI-uptake restoration.^12^

Despite these initially disappointing strategies, the first successful pioneering study in 2013 used selumetinib, a small molecule MEK1 and MEK2 inhibitor, which restored or enhanced RAI uptake in 60% of patients—marking a new era in redifferentiation therapy.^13^ This approach involves a short course of a MAPK inhibitor followed by a therapeutic activity of ^131^I. The toxicities of the approach are short-lived and reversible, which is appealing in patients with RAIR thyroid cancer with low-volume and slow-growing disease. Several additional prospective phase 2 studies with different designs have been published in the last 10 years; all but one (an adjuvant trial^14^) were not randomised (table 1), and none showed an improvement in overall survival, progression free survival, or quality of life. Despite the small numbers of patients treated within these prospective trials, the low amount of evidence of clinical benefit from this treatment approach, and the lack of regulatory agency approval, redifferentiation treatment has become a more accepted treatment strategy in patients with RAIR thyroid cancer with targetable mutations suitable for a redifferentiation approach.

Many outstanding questions remain—eg, who should be treated and when; which kinase inhibitor should be given and for how long should it be administered before RAI therapy; should dosimetry be performed and, if so, which radionuclide should be used; and what activity of ^131^I should be given for the treatment? Additionally, since all kinase inhibitors can have both an anti-tumour and a redifferentiation effect, the specific effect of the kinase inhibitors and ^131^I therapy should be defined. The objective of this Review is to examine the current evidence on redifferentiation strategies, identifying both their strengths and limitations, to provide a framework for future research directions.

### Differences in redifferentiation trial design and their potential impact on treatment efficacy

**Figure 1:**
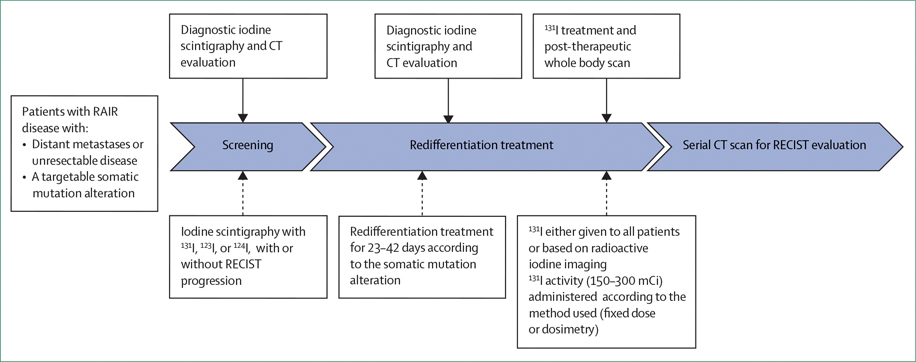
Prospective trial design RAIR=radioactive iodine refractory.

Prospective trials designed to date share many similarities. Although the details of treatment schemas vary between studies, patients with RAIR follicular cell-derived thyroid cancer harbouring a targetable gene alteration and structural non-resectable disease are treated with RAI while concurrently receiving MAPK pathway inhibitors. MAPK inhibitors are discontinued after RAI administration. The schema usually involves a diagnostic iodine scintigraphy after recombinant human thyroid-stimulating hormone (rhTSH) stimulation, a CT scan without contrast injection before initiating the MAPK inhibitor, a short duration of MAPK inhibitor treatment, a second diagnostic iodine scintigraphy after rhTSH stimulation while on the MAPK inhibitor, followed by administration of a high activity of ^131^I after another rhTSH stimulation in patients exhibiting increased iodine uptake on the second diagnostic scintigraphy. Patients are then monitored by follow-up CT imaging and serum thyroglobulin (Tg) measurements to evaluate structural and biochemical responses to redifferentiation therapy (figure 1), with the structural changes determined by Response Evaluation Criteria in Solid Tumours (RECIST).

### Inclusion criteria

The definition of RAIR disease varied between studies (table 1). All studies were performed in people older than 18 years. Patients were considered RAI refractory if one or more lesions lacked ^131^I uptake on a previous diagnostic whole body scan (ie, performed after a low activity of ^131^I ranging from 3 mCi to 5 mCi [111 MBq to 185 MBq]) or on a previous post-therapy whole body scan (ie, performed after a high activity of ^131^I whole body scan of >100 mCi [>3·7 GBq]). Most,^13–19^ but not all,^20,21^ studies included tumour progression by RECIST within 6–12 months after a previous ^131^I treatment as evidence of RAIR disease. Additionally, some trials included the presence of at least one [^18^F]fluorodeoxyglucose ([^18^F]FDG)-avid lesion with a maximum standard uptake value ( SUV_max_) of 5 or more as part of their RAIR criteria.^13–16^ The definition of RAI refractoriness remains a matter of debate. The absence of iodine uptake on a diagnostic whole body scan and the presence of [^18^F]FDG-avid lesions are risk factors for refractoriness, but some clinicians do not consider these two factors sufficient to diagnose RAIR disease.^22–24^

The inclusion criteria also differed between trials. Some studies required tumour progression within 12–18 months (per RECIST version 1.1) before study entry.^17–19^ Limits on maximum lesion size were imposed in only two studies. One study restricted lesion size to a maximum of 30 mm due to better efficacy of ^131^I treatment in smaller lesions.^1,17,18^ Another study required at least one lesion larger than 15 mm to allow for accurate dosimetry calculations, which are less precise for smaller lesions.^21^ Most studies focused on patients with thyroid cancer with *BRAF*^V600E^ (ie, Val600Glu) or *RAS* mutations, although some included a mix of different mutation types or patients with RAIR without a known mutation (table 1). Overall, most participants had papillary or follicular thyroid cancer, with only a few having poorly differentiated thyroid cancers (table 1). Finally, although previous anti-VEGF treatments were allowed, over 90% of patients were treated in the first-line setting across all trials, except for one study involving patients with *RAS*-mutated thyroid cancer, where 37% of patients were treated in the second-line setting.^17^ Because treatment is short and adverse event rates are low, redifferentiation is seen as an early line treatment that could offer the benefit of delaying the initiation of continuous therapy. However, this approach needs to be validated in clinical trials where this endpoint is prospectively evaluated.

### MAPK inhibitor treatment

MAPK activation in thyroid cancer is inversely correlated with a thyroid differentiation score, which includes genes related to thyroid function and iodine metabolism, including the genes encoding for the sodium iodine symporter (NIS; also known as SLC5A5), thyroglobulin, and thyroperoxidase.^25^ *BRAF*^V600E^-mutated and *RAS*-mutated thyroid cancers activate the MAPK pathway to different degrees. In the case of *BRAF*^V600E^ mutation, the MAPK output does not respond to the negative feedback from ERK signalling, whereas in the case of the *RAS* mutation, it does. As a result, high MAPK outputs and low thyroid differentiation scores are observed in *BRAF*^V600E^-like thyroid cancers compared with *RAS*-like thyroid cancers. In *BRAF*^V600E^ mouse models of thyroid cancer, pharmacological inactivation of MAPK signalling partially restored NIS expression and RAI avidity of the thyroid cancer.^26^ Furthermore, potent inhibition of ERK signalling is also required to adequately induce iodide uptake.^27^ This requirement has led to the use of anti-BRAF, anti-MEK, or their combination in treating patients.

The redifferentiation treatment used in the trials included small molecule inhibitors of MEK1 and MEK2 (selumetinib), *BRAF*^V600E^ (dabrafenib and vemurafenib), or a combination of a *BRAF*^V600E^ inhibitor with a MEK1 and MEK2 inhibitor (dabrafenib plus trametinib). Finally, because the inhibition of *BRAF*^V600E^ results in negative feedback—leading to the increased expression of epidermal growth factor response 3 (ERBB3) that heterodimerises with ERBB2 to induce MAPK pathway reactivation—the association of an anti-BRAF (vemurafenib) with a monoclonal antibody targeting ErbB3 (CDX-3379) was used in one trial^16^.

### Primary objectives

The primary objectives vary across trials. In most studies, the primary objective is the rate of successful redifferentiation, defined by a significant restoration of radioiodine uptake.^13–16,20,21^ The optimal definition of what constitutes as clinically relevant radioiodine uptake remains to be established, since not all radioiodine uptake will translate into tumour response. In two trials, the primary objective was tumour response rate at 6 months, assessed by RECIST version 1.1,^17,18,28^ whereas median progression-free survival was the primary objective in another study.^19^ In all cases, however, the success rate of iodine uptake restoration and tumour response rates (best partial response, 6-month partial response, or both, with or without progression-free survival) were reported.

### Definition of successful restoration of RAI uptake

The definition of significant iodine uptake restoration also differs between studies. Significant iodine uptake restoration was based on ^124^I PET–CT dosimetry by Ho and colleagues, Dunn and colleagues, and Tchekmedyian and colleagues.^13–16^ Redifferentiation was considered successful if, following MAPK inhibition, a tumour-absorbed dose of more than 20 Gy could be delivered to at least one lesion after the administration of a ^131^I activity of 300 mCi (11·1 GBq) or less.^13–16^ In the absence of ^124^I PET–CT, which is not available in many institutions, ^131^I or ^123^I whole body scans can be used to define significant iodine uptake restoration. The presence of ^131^I uptake seen in any disease site (without requiring new uptake in all known disease sites) was used by Rothenberg and colleagues.^20^ In an attempt to establish a more precise definition, high uptake in at least one tumoral lesion identified by visual assessment on a ^123^I whole body scan and single-photon-emission computed tomography (SPECT)–CT was used by Weber and colleagues with the following definition: “a regional target/background ratio of more than 4 and a 2-fold higher iodine uptake than the mean uptake in liver parenchyma in at least one tumor lesion”.^21^ The appearance of clinically relevant uptake in a lesion that had shown no uptake at baseline, or an increase of at least 30% of the baseline iodine uptake based on ^123^I whole body scan and SPECT–CT with dosimetry was used by Wadsley and colleagues.^19^ Finally, in the studies by Leboulleux and colleagues, the definition of successful iodine uptake restoration was based on the post-therapeutic ^131^I whole body scan and SPECT–CT, but the decision to proceed with ^131^I treatment was not based on the results of the diagnostic scintigraphy.^17,18^

**Figure 2:**
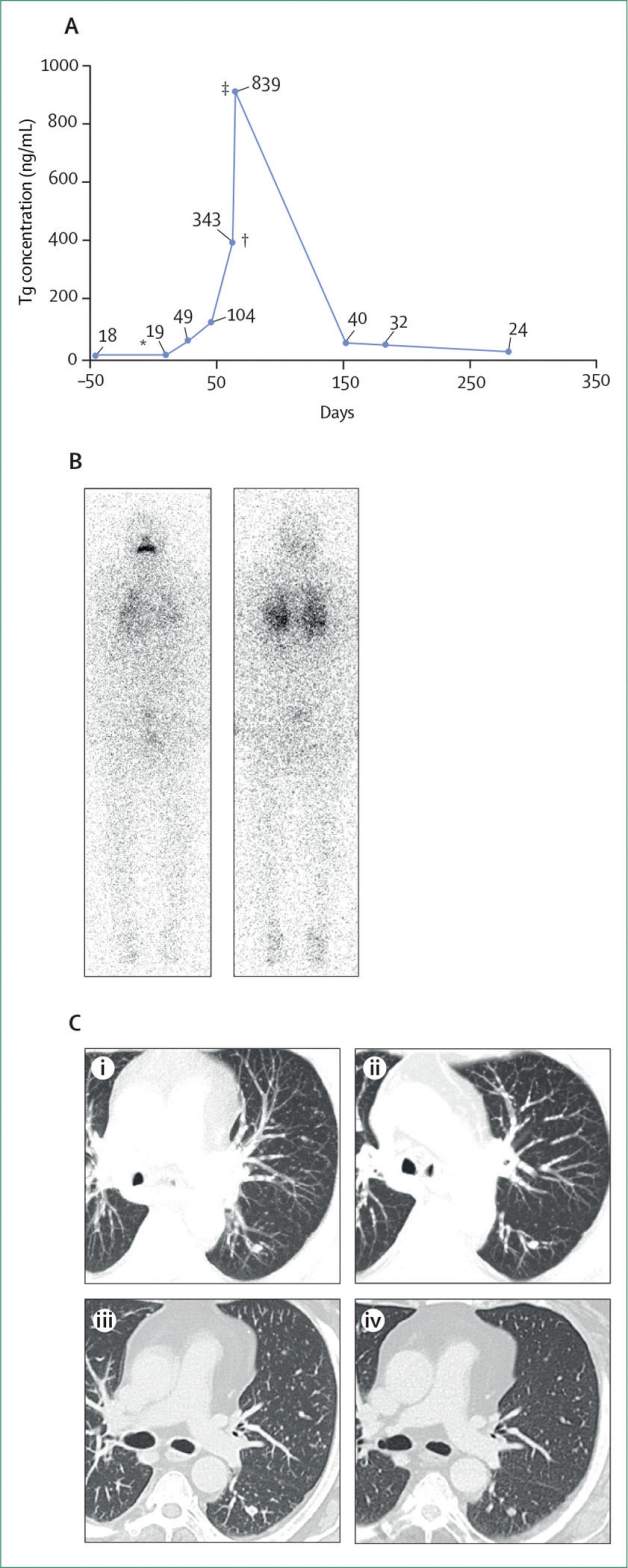
Redifferentiation therapy results in patient with *BRAF*^V600E^-mutated papillary thyroid cancer (A) Changes in Tg concentration under treatment with dabrafenib and trametinib. (B) Diffuse but low lung uptake on the post-therapeutic whole body scan after 6 weeks of dabrafenib and trametinib with ^131^I (5500 Mq) after rhTSH. (C) Follow up CT scans showing: baseline of miliary lung metastases with a 1 cm left nodule (i); stable disease after 4 weeks of dabrafenib and trametinib (ii); stable disease 3 months after 6 weeks of dabrafenib and trametinib with ^131^I (5500 Mq) after rhTSH (iii); and stable disease 6 months after 6 weeks of dabrafenib and trametinib with ^131^I (5500 Mq) after rhTSH (iv). rhTSH=recombinant human thyroid-stimulating hormone. Tg=serum thyroglobulin. *Day 0 is the start of dabrafenib and trametinib. †Day 3 after rhTSH. ‡Day 5 after rhTSH.

Overall, the rate of successful restoration of iodine uptake varies widely, ranging from 40% to 95% depending on the study (figure 2). Nonetheless, the current definitions of successful iodine uptake restoration have limited use and are not reliably predictive of therapeutic benefit. One probable reason for this limitation is that the definition for successful restoration of iodine uptake often fails to account for lesion-to-lesion heterogeneity, which introduces a high degree of subjectivity into the assessments. In addition, the MERAIODE study showed that a diagnostic ^131^I scintigraphy performed after 4 weeks of dabrafenib plus trametinib therapy did not predict tumour response at 6 months.^18^ However, establishing validated criteria for successful restoration of iodine uptake that is predictive of ^131^I efficacy would be of significant clinical value, since it would allow limiting the administration of high ^131^I activity to patients who are likely to benefit. Although ^124^I PET–CT dosimetry provides quantitative data that are more accurate than ^131^I or ^123^I scintigraphy, the issue of interlesional variability in iodine uptake is not accounted for this method and remains an unresolved problem.

### Radioiodine treatment

^131^I administration is performed under thyroid stimulating hormone (TSH) stimulation to increase iodine uptake. In non-RAIR metastatic disease, iodine uptake after recombinant human TSH (rhTSH) has been shown to be lower than that observed following thyroid hormone withdrawal.^29^ However, retrospective studies have shown no differences in survival in patients treated with rhTSH versus thyroid hormone withdrawal—with the limitation of short follow-up in patients who could have survived over 10 years.^30–34^ In all redifferentiation trials,^131^I was administered after rhTSH stimulation following treatment with MAPK inhibitors to avoid the toxicities of thyroid hormone withdrawal that could be dose-limiting in the context of treatment with MAPK inhibitors. An adverse event related to thyroid hormone withdrawal might require a dose adjustment of the redifferentiation drug even if the adverse event was not attributable to the drug itself. This, in turn, might compromise the efficacy of redifferentiation. This is important since the redifferentiating effect of dabrafenib plus trametinib was shown to be transient and to disappear within a few days in a clinical case.^35^ Furthermore, as shown in a retrospective study, exposure to the active metabolite of dabrafenib (hydroxy-dabrafenib) can correlate with successful iodine uptake restoration.^36^ Biologically, the degree of redifferentiation achieved has been shown to correlate directly with the potency of MAPK pathway inhibition imparted, leading to the clinical assumption that maintaining dose intensity and continuity is important.^27^ Nevertheless, a case report of redifferentiation therapy after withdrawal has been reported.^37^

The activity of ^131^I administered varied from study to study (table 2). Although some have used a fixed activity of 150 mCi (5·5 GBq),^17–20^ others have performed pretreatment dosimetry. The goals of dosimetry are first to search for a relationship between the radiation dose delivered to the lesion and the tumour response, and second to calculate the therapeutic activity of ^131^I to be administered to reach a sufficient radiation dose to the lesions to obtain tumour response. Dosimetry can be performed using diagnostic ^123^I scintigraphy and SPECT–CT, diagnostic or therapeutic ^131^I scintigraphy and SPECT–CT, or ^124^I PET–CT. However, the value of the absorbed doses required to obtain a tumour response and cure is still under investigation.^38^ The radiation dose delivered to the lesions depends on the iodine concentration (which takes into account the amount of iodine uptake and the tumour volume) and the residence time (which takes into account both the physical and biological half-lives of iodine).

In a study published in 1983, dosimetry in patients with non-RAIR disease showed a correlation between the dose to the tumour and the tumour response in 137 metastatic lymph nodes or distant metastases, with tumour response observed in 98% of lesions receiving more than 80 Gy and 12% of lesions receiving less than 80 Gy.^39^ More recently, ^124^I PET–CT dosimetry in patients with distant metastases (mainly located in the lungs) showed all nine lesions receiving an absorbed dose of more than 75 Gy had partial or complete response (according to modified RECIST criteria), whereas two of the ten lesions receiving less than 75 Gy showed stable disease or progression. However, the median absorbed dose of the lesions with partial or complete response was very high (194 Gy).^40^ Another study, with ^124^I PET–CT also showed that for bone lesions, which are known to be more resistant to ^131^I, an absorbed dose threshold of 85 Gy resulted in a response rate of 46%.^41^ Overall, the higher the lesional absorbed dose, the higher the probability of a therapeutic response to ^131^I therapy. However, interlesional heterogeneity is a challenge and a treatment limitation.^41^ As an example, under rhTSH stimulation, the median effective half-life of the iodine isotope in distant metastases was 21·9 h in one study but ranged from 8·7 h to 113·9 h.^29^ Consequently, from an oncological perspective, the minimal dose delivered to lesions should be used to define the activity to administer.

In general, pretreatment dosimetry in patients who are not RAI refractory aims to deliver doses to the tumour of more than 80 Gy and limiting toxicity to the bone marrow and lungs. The maximal tolerated activity is considered to be 2 Gy to the blood, based on the rate of serious haematopoietic adverse events, which was 21% for blood doses of more than 2 Gy versus 2% for blood doses of less than 2 Gy.^42^ However, the usefulness of pretreatment dosimetry in daily practice is controversial. Outside of the setting of redifferentiation, in a retrospective study, the survival of patients with distant metastases was not different when treated with radioiodine according to pretreatment dosimetry or with the administration of a fixed activity of 100 mCi (3·7 GBq) of ^131^I.^43^ Data derived from RAI sensitive tumours should be applied with caution to RAIR tumours as they could be radioresistant. As a result, a higher dose of radiation might be necessary to achieve a tumour response.

In the redifferentiation studies, the Memorial Sloan-Kettering Cancer Center dosimetry protocol aims to establish the maximal activity defined by the activity that will deliver a maximum of 2 Gy to the blood and results in a maximum of 80 mCi (2·96 GBq) 48 h-retention in the presence of diffuse lung disease or 120 mCi (4·44 GBq) 48 h-retention in the absence of diffuse lung disease.^44^ Based on the ^124^I PET–CT, patients are treated if a dose of 20 Gy can be delivered to at least one lesion with an ^131^I activity of 300 mCi (11·1 GBq) or less. With this approach, the mean ^131^I activity administered ranges from 236 mCi to 300 mCi (8·7–11·1 GBq).^15,16,21^ In the SEL-I-METRY trial, no correlation was found between pretreatment dosimetry and tumour response, but data were limited by the number of patients studied.^45^

In the context of redifferentiation, Nagarajah and colleagues described a patient in whom MAPK inhibitors increased iodine uptake, but did not affect residence time.^46^ In a prospective study, dosimetry comparing ^131^I uptake before and after selumetinib treatment, showed a 10-fold increase in the iodine uptake, with an iodine uptake of 0·2 MBq/cm^3^ (range 0·001–11·5) before treatment, and 2·1 MBq/cm^3^ (range 0·01–175·4) after treatment.^45^ Based on ^124^I PET–CT dosimetry, Weber and colleagues showed a 9·3-fold increase in the lesion-absorbed dose assessment comparing iodine uptake before and after dabrafenib plus trametinib treatment, with a median absorbed dose of 0·03 Gy/mCi (SD 0·04) before treatment and 0·28 Gy/mCi (SD 0·22) after dabrafenib plus trametinib treatment.^21^ Dosimetry performed while patients are on MAPK inhibitors before ^131^I treatment has been shown to overestimate post-therapeutic dosimetry by approximately 33% with a pre-^131^I dosimetry prediction of 17·2 Gy (range 0·11–292·zs) compared with a post-^131^I dosimetry prediction of 10·4 Gy (range 0·31–169·9).^45^ Determining the minimal absorbed dose to achieve tumour response after MAPK and high activities of ^131^I treatment is important and still needs to be defined in the redifferentiation setting, especially if dosimetry is used to define the ^131^I activity to administer.

### Tumour responses

The efficacy of the MAPK inhibitors and ^131^I treatment on tumour size was reported in all studies, with the RECIST version 1.1 response at 6 months being the primary endpoint in two trials, and the median progression-free survival in one trial.^17–19^ In patients with *BRAF*^V600E^-mutated tumour, the best partial response rate according to RECIST version 1.1 ranges from 0% to 57%, and the 6-month partial response rate was 33% and 38%, respectively, in two studies (table 3).^13–16,20,21^ Tumour shrinkage in response to the MAPK inhibitor might begin before ^131^I is administered, raising questions about the contribution to efficacy of the MAPK inhibitor alone versus the MAPK inhibitor plus ^131^I.^15,17^ One hypothesis is that tumours most sensitive to these agents will both show a direct anti-tumour effect and a high degree of redifferentiation, and the final result (in terms of RECIST tumour response) might be unrelated to ^131^I administration.

In the MERAIODE study, the best partial response rate occurred at 3 months (57%) whereas the 6-month partial response rate was 38%. In patients who had progressed according to RECIST version 1.1 within 18 months before enrolment, the 12-month and 24-month progression-free survival rates were 82% and 68%, respectively. In comparison, the 12-month progression-free survival rate was 65% in the SEL-I-METRY trial, which used selumetinib to treat patients with agnostic RAIR thyroid cancer who had progressed within 12 months before enrolment.^19^

Fewer studies have been reported in patients with *RAS-*mutated thyroid cancer (table 3). In a study with five patients treated with selumetinib, all patients showed iodine uptake restoration and were treated with high activity of ^131^I. Four of these five patients showed a partial response, which is very encouraging.^13^ In another study of ten patients treated with trametinib, only 60% of the patients had iodine uptake restoration. All patients were treated with a high activity of ^131^I. The 6-month partial response rate, which was also the best partial response, was only 20%, in contrast to the first study.^17^ Preliminary results from a third study of 25 patients treated with trametinib showed successful iodine uptake restoration in 88% of patients with a 6-month response rate of 32%.^28^

Only one trial assessed the efficacy of a second course of dabrafenib plus trametinib followed by administration of ^131^I in patients with *BRAF*^V600E^ mutated thyroid cancer. There were 11 patients who started the second course of treatment and ten were evaluable. The median decrease in lesion size was 53% as compared with before study entry, but no change as compared with the baseline of the second course. Thus, the added effect of this second course of treatment was very low.^18^ However, it is important to note that the number of patients enrolled in each of these studies remains small.

Time to retreatment has also been considered as a potential criterion to evaluate the benefit of redifferentiation, especially when the latter is used as first-line therapy.^17,18^ However, the criteria for initiation of treatment with kinase inhibitors are not uniform and are subjective, as they depend on tumour burden, tumour growth rate, symptoms, patients’ comorbidities, and patient preference,^47–51^ which makes this an imprecise marker of clinical benefit. Other ongoing clinical trials should provide more information on the possible benefit of this redifferentiation strategy (NCT06440850, NCT05182931, NCT04619316, and NCT04858867; appendix).

### Safety

The short duration of MAPK inhibition has the advantage of reducing adverse events. However, more than 80% of patients suffer at least one adverse event, with grade 3 adverse events ranging from 5% to 46%, and grade 4 adverse events being less than 5% (table 4).^13–21^ Furthermore, the rate of discontinuation of MAPK inhibitors is high, ranging from 5% to 21% and even reaching 55% in one study.^16^ With dabrafenib and trametinib, most patients had fatigue, nausea, vomiting, diarrhoea, fever, rash, and decreased appetite. Quality of life, as measured by QLQC30 questionnaires, was shown to be reduced after initiation of dabrafenib plus trametinib with a decrease in four of the five functional dimensions: physical, role, cognition, and social.^18^ This decrease in quality of life, which persisted for a median of 42 days (ie, the duration of treatment with MAPK inhibitors), was reversible, with scores returning to baseline after MAPK inhibitors were stopped. Similarly, the adverse events were temporary and resolved after treatment with MAPK inhibitors was stopped. The optimal duration of the therapy has not been explored and, thus, shorter courses of treatment could be effective while limiting toxicity.

### Prediction of successful redifferentiation

Predictors of RAI refractoriness studied include [^18^F] FDG uptake, Tg levels, tumour shrinkage under redifferentiation agents, diagnostic scintigraphy, and genetics. Low [^18^F]FDG uptake was found to be a predictive factor for successful iodine uptake restoration, with none of the patients with a SUV_max_ of more than 10 exhibiting successful iodine-uptake restoration.^21^ However, in another study, [^18^F]FDG uptake intensity at baseline was not predictive of the 6-month tumour response.^18^

The unstimulated pre-redifferentiation baseline Tg concentration was also found to be predictive of iodine uptake restoration, with no patient with thyroglobulin of less than 66 ng/mL showing successful iodine uptake-restoration whereas 50% of patients with Tg of more than 66 ng/mL showed successful iodine uptake restoration.^21^ Unstimulated Tg concentrations are known to increase in response to MAPK inhibitors in the absence of tumour progression—an effect that is reversible when the treatment is stopped.^15,35^ This effect is important to emphasise because in this situation treatment should not be discontinued based on Tg increase alone, as in this setting, Tg levels are a biomarker of redifferentiation. Whether an increase of Tg is a better predictor of successful treatment (iodine uptake restoration and tumour response) will need to be assessed in the future.

Tumour shrinkage in redifferentiation studies started while patients were on MAPK inhibitors, before ^131^I administration, with RECIST partial response being reached in 20% of the patients with *BRAF*^V600E^*-*mutated thyroid cancer treated with vemurafenib, 48% of the patients with *BRAF*^V600E^*-*mutated thyroid cancer treated with dabrafenib plus trametinib, and 10% of the patients with *RAS*-mutated thyroid cancer treated with trametinib.^15,17,18^ In patients with *BRAF*^V600E^-mutated thyroid cancer treated with dabrafenib plus trametinib, the 1-month RECIST tumour response did not correlate with the 6-month tumour response. 50% of the patients who reached a 1-month RECIST partial response remained in partial response at 6 months, whereas 27% of patients who did not achieve a 1-month RECIST partial response achieved partial response at 6 months (p=0·38).^18^

Finally, many studies have used a diagnostic ^123^I or ^131^I whole body scan performed after treatment with MAPK inhibitors to determine if ^131^I should be administered, with subjective interpretation of what is defined as successful iodine uptake restoration. However, in patients with *BRAF*^V600E^-mutated thyroid cancer treated with dabrafenib plus trametinib, the diagnostic ^131^I whole body scan performed during treatment was not predictive of the response at 6 months. In this context, 37% of patients with increased ^131^I uptake on the diagnostic whole body scan had a partial response at 6 months compared with a 33% partial response in patients without ^131^I uptake.^18^ This observation suggests that the absence of ^131^I uptake on a whole body scan might not be predictive of the lack of benefit in all cases. The low sensitivity of a diagnostic whole body scan might be an explanation as the patients did have abnormal uptake on the post-therapeutic whole body scan. However, we cannot exclude a long-term benefit of treatment with MAPK inhibitors or an effect of the repeated rhTSH stimulation at short-term interval. Patients with structural disease and negative diagnostic scintigraphy were shown not to benefit from high activities of ^131^I in terms of tumour response, despite evidence of ^131^I-avid lesions on post-therapeutic scintigraphy.^52^

Co-mutations in the SWI/SNF complex have been shown to cause resistance to redifferentiation therapy—with the loss of individual SWI/SNF subunits leading to a repressive chromatin state that cannot be reversed by blockade of the MAPK pathway—rendering them insensitive to redifferentiation. ^53^ Three patients with a mutation in the SWI/SNF complex who had no iodine uptake restoration after MAPK inhibitor therapy were reported. However, there was also one patient with a *BRAF*^V600E^ mutation and a *SMARCA4* mutation (part of the SWI/SNF complex) who was treated with dabrafenib plus trametinib, and had a 6-month partial response.^18^ Other mutations enriched in poorly differentiated or anaplastic thyroid cancer associated mutations, such as *TP53* or *PIK3CA* could also reflect biology refractory to differentiation, although more validations of these alterations as predictive biomarkers are needed. Finally, besides this primary resistance to redifferentiation that can be defined by the absence of iodine uptake restoration, one can also define resistance related to the absence of tumour response after the first or even the second course of treatment of redifferentiation agents and ^131^I treatment.

### Redifferentiation in real-world data

Real-world data typically show worse results compared with prospective trials, as many patients included in real-world analyses would not meet the inclusion criteria of the prospective trials.^54^ In redifferentiation trials, one study showed 37% iodine uptake restoration and 6% best partial response rate for *BRAF*^V600E^-mutated thyroid cancer, and 82% iodine uptake restoration and 8% best partial response rate for *RAS*-mutated thyroid cancer.^55^ Patients treated in the study had more advanced disease, with a 15% death rate after a median 24-month follow-up. These results show the importance of patient selection when investigating redifferentiation therapy. Real-world data also raised the question of a possible link between redifferentiation treatments and anaplastic transformation, with two of the 33 patients treated experiencing anaplastic transformation after redifferentiation.^55^ However, this finding will need to be further scrutinised. Of note, the relationship between iodine treatment and anaplastic transformation was raised in the 1970s when treatment with ^131^I started to be widely used, without being subsequently confirmed.^56^

### Redifferentiation for other genotypes

Most prospective redifferentiation studies have included patients with *BRAF*^V600E^ and *RAS-*mutated tumours. The first study by Ho and colleagues enrolled three patients with a RET/PTC fusion thyroid cancer, one of whom had sufficient iodine uptake restoration to receive a high activity of ^131^I after selumetinib treatment,^13^ with the best response being stable disease. In a retrospective study on redifferentiation, one of the three patients with RET/PTC fusions showed successful iodine uptake restoration, but this restoration did not achieve a partial response.^55^ By contrast, successful iodine uptake restoration has been reported in three patients with RET/PTC fusions treated with the selective RET kinase inhibitor selpercatinib,^57–59^ two of whom achieved tumour responses. However, given the high efficacy of selpercatinib in RET/PTC thyroid cancer, it is difficult to know whether the efficacy is related to the selpercatinib itself or to the combination of selpercatinib and ^131^I therapy.^60^ In addition, systemic treatment was maintained long-term after ^131^I treatment. Clinical cases of both successful and unsuccessful iodine uptake restoration have been reported in patients with *NTRK* fusion thyroid cancer treated with larotrectinib.^58,61,62^ Prospective multi-centre redifferentiation trials with selpercatinib or larotrectinib and ^131^I are ongoing, and should yield informative data (NCT05668962, NCT06458036, and NCT05783323; appendix). Other genotypes are also logical candidates for redifferentiation approaches, including *ALK* fusions and *NF1* loss-of-function mutations.^63^

### Redifferentiation in the future

The amount of evidence supporting the use of redifferentiation strategies for treating patients with RAI refractory disease is low. There are no randomised studies showing an improvement in overall survival or progression-free survival, and no comparison to the standard of care. Cumulative data to date on redifferentiation indicate a moderate amount of evidence of clinical benefit. Encouraging RECIST version 1.1 response rates and median progression-free survival have been reported, but the number of patients included in the phase 2 trials is small. So far, only one complete response has been reported.^18^ Redifferentiation therapy for patients with RAIR disease, therefore, remains palliative, which should be taken into account when considering treatment for patients with low tumour burden and indolent disease, especially if treatment is given as first-line.

More evidence of the efficacy and long-term benefits of redifferentiation therapy is needed. Ideally, future trials should address the following: the respective impacts of a short course of kinase inhibitor treatment versus the kinase inhibitor with ^131^I treatment on response to therapy, the role of dosimetry, and the optimal length of redifferentiation treatment. In addition, trials should address which patients are most likely to benefit from a redifferentiation strategy—in terms of disease progression rate before treatment, disease burden, [^18^F] FDG uptake, molecular alterations—and thus be ideal candidates. Studies should investigate the most effective redifferentiation agent based on the specific somatic genetic alteration present in each patient and the efficacy of subsequent treatment if redifferentiation is given as first-line treatment. Peripheral markers documenting the extent of suppression of the targeted pathway should also be further explored.

## Conclusion

The efficacy of redifferentiation strategies should not only evaluate the proportion of patients with increased iodine uptake, but also the tumour response rate after MAPK inhibitor treatment alone, the 3-month and 6-month response rate after the combined MAPK inhibitor with ^131^I therapy, as well as the progression-free survival. Time to retreatment is a subjective criterion and should be used with caution. Finally, the optimal time to initiate redifferentiation has not been elucidated and remains controversial. We strongly favour treating patients in the context of clinical trials, but recognise that redifferentiation is being widely used off-label. Substantial insight will emerge from experiences reporting the use of redifferentiation agents outside of clinical trials. In these cases, thorough characterisation of patients will be essential, and the evaluation of efficacy should encompass not only rates of iodine uptake restoration, but also structural tumour response to therapy.

## Supplementary Material

MMC1

## Figures and Tables

**Table 1:** Inclusion criteria and patient characteristics

	Patients included (patients evaluated)	Inclusion criteria	Type of mutation (number)	PTC	FTC	PDTC	First-line treatment	Second-line treatment

Ho et al (2013)^13^	24 (20)	RAIR disease defined by (a) a non-RAI-avid metastatic lesion on a diagnostic whole body scan <2 years before enrolment, or (b) an RAI-avid lesion stable or progressive in size despite therapeutic ^131^I >6 months before study entry, or (c) at least one [^18^F]FDG-avid lesion with SUV_max_ ≥5; and measurable disease according to RECIST; and at least one [^18^F]FDG-avid lesion	*BRAF*^V600E^ 9; *NRAS* 5; *RET/PTC* 3; wild-type 3	13	0	7	18	2*
Rothenberg et al (2015)^20^	10 (10)	Somatic *BRAF*^V600E^ mutation and metastatic or unresectable disease; and no ^131^I uptake on a whole body scan (diagnostic or post treatment) within 14 months of study entry; and evaluable by CT scan; and ECOG ≥1	*BRAF*^V600E^ 10	10	0	0	10	0
Dunn et al (2019)^15^	12 (10)	BRAF^V600E^ mutation and RAIR disease defined by (a) a non-RAI-avid metastatic lesion on a diagnostic WBS <2 years before enrolment, or (b) an RAI-avid lesion of stable size or which progressed despite therapeutic ^131^I >6 months before study entry, or (c) at least one [^18^F]FDG-avid lesion with SUV_max_ ≥5; and no ^131^I treatment <6 months before study entry	*BRAF*^V600E^ 12	9	0	3	11	1†
Tchekmedyian et al (2022)^16^	7 (7)	*BRAF*^V600E^ mutation and RAIR disease defined by (a) a non-RAI-avid metastatic lesion on a diagnostic whole body scan <2 years before enrolment, or (b) an RAI-avid lesion of stable size or an RAI-avid lesion that progressed despite therapeutic ^131^I >6 months before study entry, or (c) at least one [^18^F]FDG-avid lesion with SUV_max_ ≥5; and measurable disease according to RECIST	*BRAF*^V600E^ 7	4	0	3	7	0
Weber et al (2022)^21^	20 (20)	Unresectable or RAIR disease, or both; or RAIR disease defined by at least one metastatic lesion without therapeutically sufficient RAI accumulation on a diagnostic or therapeutic RAI scan performed less than 1 year before enrolment, or both; and at least one lesion ≥15 mm (short-axis diameter for nodes; long-axis diameter for other lesions)	*BRAF*^V600E^ 6; *NRAS* 2; *KRAS* 1; *APC* 1; *AKT* 1; *PTEN* 3; no mutation 6	10	7	3	20	0
Leboulleux et al (2023)^18^	24 (21)	BRAF^V600E^ mutation and RAIR disease defined by (a) presence of distant metastases without ^131^I uptake on a post-therapy whole body scan, or (b) distant metastases disclosing RECIST version 1.1 progression within 12 months after a therapeutic ^131^I administration, or both; and patients had measurable disease according to RECIST version 1.1 criteria; and no lesion >3 cm; and RECIST version 1.1 progression within 18 months before enrolment	*BRAF*^V600E^ 21; unknown 4	24	0	0	20	1‡
Leboulleux et al (2023)^17^	11 (10)	*RAS* mutation and RAIR disease defined by (a) the presence of distant metastases without ^131^I uptake on a post-therapy whole body scan, or (b) distant metastases disclosing RECIST version 1.1 progression within 12 months after a therapeutic ^131^I administration, or both; and patients had measurable disease according to RECIST version 1.1 criteria; and no lesion >3 cm; and RECIST version 1.1 progression within 18 months before enrolment	*RAS* 11	4	2	5	8	3§
Wadsley et al (2023)^19^	28 (28)	RAIR disease defined by (a) the presence of a measurable non-RAI avid lesion on a previous whole body scan, or (b) at least one measurable lesion had progressed within 12 months of RAI therapy, or both; and RECIST version 1.1 progression within 12 months before enrolment	NA	11	17	0	28	0

Data are number of patients unless specified otherwise. Inclusion criteria for thyroid cancer of follicular origin. FDG=[^18^F]fluorodeoxyglucose. FTC=follicular thyroid cancer. NA=not applicable. PDTC=poorly differentiated thyroid cancer. PTC=papillary thyroid cancer. RAI=radioactive iodine. RAIR=radioactive iodine refractory. RECIST=Response Evaluation Criteria in Solid Tumours. SUX_max_=maximum standard uptake value.

* Sorafenib plus mammalian target of rapamycin complex 1 inhibitor (two patients).

† Sorafenib (one patient).

‡ Pazopanib (one patient).

§ Pazopanib (two patients) and lenvatinib (one patient).

**Table 2:** ^131^I administration methods

	Drug	Length of treatment (days)	^131^I administration based on	Number of patients treated with ^131^I (number evaluable)	^131^I activity determination	Mean (range) ^131^I activity (mCi)

Ho et al (2013)^13^	Selumetinib	35	^124^I PET–CT; a dose ≥20 Gy is delivered to ≥1 metastatic lesion with a maximum activity of ^131^I of 300 mCi	1 (9)	^131^I and ^124^I dosimetry protocol	NA
Dunn et al (2019)^15^	Vemurafenib	35	^124^I PET–CT; a dose ≥20 Gy is delivered to at ≥1 metastatic lesion with a maximum activity of ^131^I of 300 mCi	4 (12)	^131^I and ^124^I dosimetry protocol	253 (130–401); 47–93% of the MTA
Tchekmedyian et al (2022)^16^	Vemurafenib and anti-Erb3 monoclonal antibody	35	^124^I PET–CT; a dose ≥20 Gy is delivered to at ≥1 metastatic lesion with a maximum activity of ^131^I of 300 mCi	4 (7)	^131^I and ^124^I dosimetry protocol	236 (197–299); 30–97% of the MTA
Rothenberg et al (2015)^20^	Dabrafenib	42	^131^I whole body scan with 4 mCi or rhTSH; ^131^I uptake seen in any abnormal site	6 (10)	Fixed activity	150 (150–150)
Weber et al (2022)^21^	Dabrafenib and trametinib	24	^123^I whole body scan and SPECT-CT visual assessment	2 (6)	^124^I PET dosimetry	30 (273–421)
Leboulleux et al (2023)^18^	Dabrafenib and trametinib	42	All patients received therapeutic ^131^I regardless of the results of the diagnostic ^131^I scintigraphy	20 (21)	Fixed activity	150 (150–150)
Leboulleux et al (2023)^17^	Trametinib	42	All patients received therapeutic ^131^I regardless of the results of the diagnostic ^131^I scintigraphy	11 (10)	Fixed activity	150 (150–150)
Wadsley et al (2023)^19^	Selumetinib	28	^123^I whole body scan and SPECT-CT with dosimetry; clinically relevant uptake if no uptake at baseline, ≥30% increase in RAI uptake if present at baseline	11 (28)	Fixed activity	150 (150–150)

MTA=maximal tolerated activity. NA=not applicable. rhTSH=recombinant human thyroid-stimulating hormone. SPECT-CT=single photon emission CT.

**Table 3:** Tumour response in thyroid cancer with *BRAF*^V600E^ or *RAS* mutations

	Treatment	Number of patients included (number evaluable)	Iodine uptake evaluation method	Number of patients with increased iodine uptake	Number of patients treated with ^131^I	Number of patients with complete response	Partial response

***BRAF*^V600E^ mutation**							
Ho et al (2013)^13^	Selumetinib with or without ^131^I	NA (9)	^124^I PET–CT	4 (44%)	1	0	11% (best)
Rothenberg et al (2015)^20^	Dabrafenib with or without ^131^I	10 (10)	Diagnostic ^131^I whole body scan	6 (60%)	6	0	20% (best)
Dunn et al (2019)^15^	Vemurafenib with or without ^131^I	12 (10)	^124^I PET–CT	6 (60%)	4	0	20% (best)
Tchekmedyian et al (2022)^16^	Vemurafenib and anti-ErbB3mAb with or without ^131^I	7 (6)	^124^I PET–CT	5 (80%)	4	0	33% (6 months)
Weber et al (2022);^21^	Dabrafenib and trametinib with or without ^131^I	6 (6)	^124^I PET–CT	2 (33%)	6	0	0% (best)
Leboulleux et al (2023)^18^	Dabrafenib and trametinib with or without ^131^I	21 (24)	Post-treatment ^131^I whole body scan	20 (95%)	21	1	38% (6 months); 57% (best)
***RAS* mutation**							
Ho et al (2013)^13^	Selumetinib with or without ^131^I	NA (5)	^124^I PET–CT	5 (100%)	5	0	80% (best)
Leboulleux et al (2023)^17^	Trametinib with or without ^131^I	11 (10)	post-treatment whole body scan	6 (60%)	10	0	20% (6 months and best)
Burman et al (2022)^28^	Trametinib with or without ^131^I	NA (25)	^124^I PET–CT	22 (88%)	15	0	32% (6 months)

Data are RECIST tumour response.

**Table 4:** Safety of MAPK inhibitor treatment followed by ^131^I administration in prospective trials

	Treatment	Percentage of patients with at least one adverse event	Percentage of patients with grade 3 adverse event (number of patients with events/number of patients treated)	Percentage of patients with grade 4 adverse event (number of patients with events/number of patients treated)	Treatment withdrawal

Ho et al (2013)^13^	Selumetinib with or without ^131^I	NA	0% (0/24)	0% (0/24)	Temporary 0/24; definitive 0/24
Rothenberg et al (2015)^20^	Dabrafenib with or without ^131^I	100%	0% (0/10)	0% (0/10)	Temporary 0/10; defintive 0/10
Dunn et al (2019)^15^	Vemurafenib with or without ^131^I	NA	NA*	0% (0/12)	Temporary 2/12 (17%); definitive 0/24
Tchekmedyian et al (2022)^16^	Vemurafenib and anti-ErbB3 with or without ^131^I	NA	57% (4/7)	0% (0/7)	Temporary 3/7 (43%); definitive 1/7 (14%)
Weber et al (2022)^21^	Trametinib with or without dabrafenib, with or without ^131^I	85%	5% (1/20)	5% (1/20)	Temporary 1/20 (5%); definitive 0/20
Leboulleux et al (2023)^18^	Dabrafenib and trametinib with ^131^I	96%	25% (6/24)	4% (1/24)	Temporary 3/24 (12%); definitive 2/24 (8%)
Leboulleux et al (2023)^17^	Trametinib with ^131^I	82%	18% (2/11)	0% (0/11)	Temporary 0/11; definitive 2/11 (18%)
Wadsley et al (2023)^19^	Selumetinib with or without ^131^I	100%	13/28 (46%)	1/28 (4%)	NA

Data are % or n/N (%). NA=not applicable.

* Five grade 3 adverse events in 12 patients.

## Search strategy and selection criteria

References for this Review were identified through searches of PubMed for articles in English and French, published between Jan 1, 1999, and Nov 15, 2024. The search terms were: “thyroid cancer”, “radioactive iodine refractory”, “redifferentiation”. This Review is not a systematic review, but we selected pertinent original articles on redifferentiation therapy.
