# Supplementary material for: Redifferentiation therapy in unresectable or metastatic radioactive iodine refractory thyroid cancer: an International Thyroid Oncology Group statement
Source: Lancet Diabetes Endocrinol. Author manuscript; Available in PMC 2026 Mar 24. (PMC13011886; doi:10.1016/S2213-8587(25)00064-6)
Supplement: MMC1 [file NIHMS2148892-supplement-MMC1.pdf]

THE LANCET
Diabetes & Endocrinology

Supplementary appendix

This appendix formed part of the original submission and has been peer reviewed. We post it as supplied by the authors.

Supplement to: Leboulleux S, Boucai L, Busaidy N, et al. Redifferentiation therapy in unresectable or metastatic radioactive iodine refractory thyroid cancer: an International Thyroid Oncology Group statement. *Lancet Diabetes Endocrinol* 2025; published online April 30. https://doi.org/10.1016/S2213-8587(25)00064-6.

Supplementary appendix

Ongoing Trials on redifferentiation (clinicaltrials.gov)

NCT Number	Title of the study	Design	Place of the Principal Investigator	Primary Objective
NCT06440850	Vemurafenib and Cobimetinib for the Treatment of Patients With High Risk Differentiated Thyroid Carcinoma With *BRAFV600E* Mutation	Single Arm Phase II	City of Hope Medical Center, USA	The proportion of *BRAF* mutated high-risk differentiated thyroid carcinoma patients who achieve excellent or indeterminate response with vemurafenib and cobimetinib treatment prior to initial radioactive iodine (RAI) therapy as defined by American Thyroid Association guideline.
NCT06458036	Selpercatinib Pre-RAI in Patients With *RET* Fusion Thyroid Cancer (RAISE)	Single Arm Phase II	Children's Hospital of Philadelphia, USA	Determine the overall, pulmonary, structural, and biochemical response rate to selpercatinib in patients with *RET* fusion differentiated thyroid cancer treated with 6 months of selpercatinib prior to 131I therapy
NCT05783323	Larotrectinib to Enhance RAI Avidity in Differentiated Thyroid Cancer	Single Arm Phase II	Children's Hospital of Philadelphia, USA	Determine the overall, pulmonary, structural, and biochemical response rate to larotrectinib in patients with *NTRK* fusion differentiated thyroid cancer treated with 6 months of selpercatinib prior to 131I therapy
NCT05668962	Restor. I-131 Upt. + Selpercatinib in RET F-P RAI-R TC	Single Arm Phase II	Massachusetts General Hospital, USA	Best overall response (ORR) (CR and PR) at 6 months.
NCT05182931	A Prospective, Multi-Centre Trial of TKI Redifferentiation Therapy in Patients With RAIR Thyroid Cancer (I-FIRST Study)	Single Arm Phase II	John Cancer Research Institute, USA	Progression free survival as assessed by RECIST 1.1 criteria at 6 and 12 months in participants who proceed to I131 treatment
NCT04619316	Enhancing Radioiodine Incorporation Into Radio Iodine Refractory Thyroid Cancers With MAPK Inhibition (ERRITI)	Single Arm Phase II	Essen, Germany	Proportion of patients with sufficiently increased tumoral iodine incorporation At the time point of 123I whole-body scintigraphy, 3 weeks after the start of redifferentiation therapy
NCT04858867	Reinducing Radioiodine-sensitivity in Radioiodine-refractory DTC Using Lenvatinib (RESET)	Single Arm Phase II	Leiden, Netherlands	Fraction of RAI-R thyroid cancer patients who are eligible for I-131 therapy after 6- or 12-week lenvatinib treatment
